# A Text-Book of Materia Medica, Therapeutics and Pharmacology

**Published:** 1903-02

**Authors:** 


A Text-Book of Materia Medica, Therapeutics and Pharmacology.—By George F. Butler, Ph. G., Professor of Materia Medica in the College of Physicians and Surgeons, Chicago, Medical Department of the University of Illinois, etc. Fourth edition, thoroughly revised. Handsome octavo volume of 896 pages; illustrated. Philadelphia and London: W. B. Saunders & Co. 1902. Cloth, $4.00, net; sheep or half morocco, $5.00, net.

This fourth edition has been thoroughly revised. The subject is presented in a most interesting and comprehensive manner, dealing more with facts than theory. The subject matter is so arranged as to be adequately descriptive, yet not voluminous, well adapted to the needs of the student as well as the practitioner of medicine.

The chapter on organ-therapy, serum-therapy and cognate subjects has been decidedly enlarged, dealing with the recent advancements, mode of administration and dosage. But perhaps the most important addition is the chapter on the newer theories of electrolytic discussion and its relation to the topic of pharmacotherapy and the relevant discussion added to the simple relations of chemical structure to drug action. T. P. L.

